# Network Pharmacology Analysis and Experimental Validation to Explore the Anti-inflammatory Mechanism of Asiatic Acid on Alcoholic Steatohepatitis

**DOI:** 10.1155/2022/1708030

**Published:** 2022-08-08

**Authors:** Tao Jiang, Jinhong Xu, Yang Lu, Xiaojin Chen, Yongxia Li

**Affiliations:** Navy Anqing Hospital, China

## Abstract

**Objective:**

The mechanism of action of asiatic acid (AA) on alcoholic steatohepatitis (ASH) was investigated using network pharmacology and experiments.

**Methods:**

Through data retrieval, network construction, and enrichment analysis, the potential mechanism of AA in the treatment of alcoholic steatohepatitis was explored. Animal and cell models were established in this study. *Animal Model*. The mouse model was divided into six groups: normal group; model group; low, medium, and high AA group; and silibinin-positive group. *Cell Model*. An *in vitro* inflammatory model of RAW264.7 cells was established by alcohol stimulation.

**Results:**

Compared with the model group, the low, medium, and high AA group showed decreased serum aspartate aminotransferase (AST), alanine aminotransferase (ALT), triglyceride (TG), and total cholesterol (T-CHO). The inflammatory factor tumor necrosis factor alpha (TNF-*α*), interleukin 1 beta (IL-1*β*), and interleukin 6 (IL-6) in a dose-dependent manner were decreased. In addition, hematoxylin-eosin staining showed that liver tissue damage and inflammatory cell infiltration in mice were significantly reduced with increasing doses. Further, oil red staining showed that lipid accumulation in hepatocytes in the low, medium, and high AA group was significantly reduced, with increasing dose. In addition, in the cellular model, real-time reverse transcriptase-polymerase chain reaction (Real-Time RT-PCR) and enzyme-linked immunosorbent assay (ELISA) results showed that AA could alleviate alcohol-induced cellular inflammation, while western blot and immunofluorescence results showed that AA might alleviate alcohol-induced cellular inflammation by inhibiting the nuclear factor-*κ*B (NF-*κ*B) pathway.

**Conclusion:**

This study provides multiple lines of evidence that asiatic acid may alleviate alcoholic hepatitis in mice by modulating the NF-*κ*B pathway.

## 1. Introduction

Alcoholic hepatitis is a liver disease caused by prolonged and excessive alcohol consumption. It is clinically characterized by nausea, vomiting, jaundice, hepatomegaly, and tenderness and can be further complicated by clinical symptoms such as liver failure and upper gastrointestinal bleeding [1–3]. Alcoholic hepatitis is the key link in the development of alcoholic liver disease. As the disease progresses, it can develop into liver fibrosis and cirrhosis, and without intervention, liver cell necrosis can be induced, leading to liver failure [4, 5]. Currently, complete abstinence is the most effective approach for treatment of alcoholic hepatitis. Although anti-inflammatory and hepatoprotective drugs have been shown to be effective in animal experiments, the results of large-scale clinical trials are still lacking [4, 6]. In view of the complex pathogenesis of alcoholic hepatitis, there is currently no anti-inflammatory or hepatoprotective drug with confirmed efficacy that can be recommended for the treatment of alcoholic hepatitis [7]. In recent years, more and more Chinese herbal extracts are recognized for their efficacy in treating diseases, and various studies have been conducted to validate the efficacy of many natural compounds in alcoholic liver disease (ALD).

Asiatic acid (AA) is a triterpene acid extracted from *Centella asiatica*. There are many literature reports on its anti-inflammatory, antitumor, and antidiabetic properties [8–11]. Specifically, its anti-inflammatory properties are very promising [12, 13]. Studies have found that AA can limit liver fibrosis in rats, but there are few reports on the effect of AA in alcoholic hepatitis.

As a classic inflammatory signaling pathway, the NF-*κ*B signaling pathway has been shown to be involved in response to inflammation in various diseases. The NF-*κ*B signaling pathway includes a family of nuclear transcription factors and is the main inflammatory pathway activated during the progression of liver injury [14, 15].

**Figure 1:**
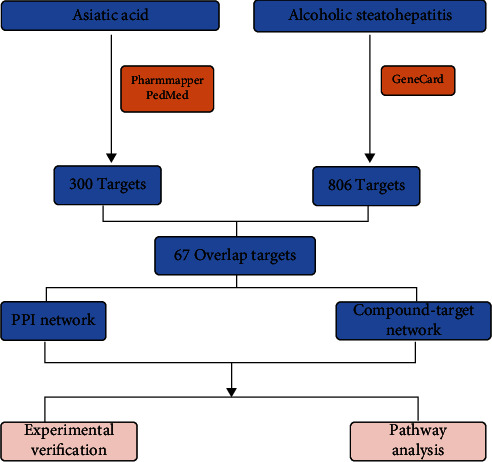
Research flow.

Network pharmacology is a relatively new discipline based on the theory of systems biology, which analyzes the network of biological systems and selects specific signal nodes to design multitarget drug molecules [16]. Network pharmacology emphasizes the multiway regulation of signaling pathways and focuses on drug research and provides new ideas, especially for the research of traditional Chinese medicine based on complex systems. [17] It provides new scientific and technical support for the rational clinical use of drugs and drug development. Growing evidence suggests that network pharmacology can elucidate the underlying mechanisms of multicomponent and multitargeted drugs by analyzing various complex and multilayered interaction networks [16, 18]. Does AA reduce alcoholic hepatitis in mice by modulating NF-*κ*B signaling? To answer this question, we first established *in vitro* and *in vivo* models and predicted the potential targets of AA in the treatment of alcoholic steatohepatitis through network pharmacology and further explored the role and mechanism of AA in mice with alcoholic hepatitis through experimental verification (Figure 1).

## 2. Materials and Methods

### 2.1. Prediction of Potential Targets of AA for the Treatment of ASH

The target information for AA was obtained from the PharmMapper database and the PubMed database [19], and duplicates were removed by integrating UniProt database entries. Using “alcoholic steatohepatitis” as the keyword, ASH-related targets were collected from the GeneCards database [20]. The AA targets and ASH-related targets were crossed to obtain the potential targets of AA for the treatment of ASH, and the AA targets and ASH-related targets were imported into VENNY2.1 for visual display.

### 2.2. Potential Target Analysis

The obtained potential targets of AA in the treatment of ASH were imported into the interactive gene/protein search tool database to obtain a protein-protein interaction (PPI) network map [21]. The minimum required interaction score was set to 0.4. The TSV format file was downloaded from the String database and imported into Cytoscape 3.8.0 for core target screening.

### 2.3. Mock Molecular Docking

We downloaded the chemical structure of AA from the PubChem database. We also downloaded the three-dimensional structure of the NF-*κ*B P65 from the protein database. Finally, Autoduck was used to perform molecular docking [22]. The binding activity of AA to NF-*κ*B P65 was evaluated according to the size of the binding energy. PyMOL software was used to visualize the docking results of AA and NF-*κ*B P65.

### 2.4. Animal Model

Female C57BL/6J mice (age: 6–8 weeks, weight: 18–22 g) were purchased from Jinan Pengyue Laboratory Animal Breeding Co., Ltd. (Jinan, China). This study was approved by the Ethics Committee of Navy Anqing Hospital. Lieber-DeCarli low-fat alcohol liquid feed and control liquid feed (TP4031 series) were purchased from Nanjing Nutrition Animal Feed High-Tech Co., Ltd. First, animals were randomly divided into the following six groups: control liquid feed group; alcohol feed group; alcohol-fed group+AA (low, medium, and high) group; and alcohol diet+silibinin-positive group, with eight animals in each group. The entire modeling period was 16 days, and the mice were weighed every 2 days. Referring to the literature [23, 24], the specific modeling method is as follows: First, all mice were fed a Lieber-DeCarli control liquid diet for 5 days to adapt to the liquid diet. At the end of the adaptation period, except the control group, all groups other than the control group were fed Lieber-DeCarli low-fat alcohol liquid feed containing 5% (vol/vol) ethanol for 10 days. From the 8th to the 15th day, AA (Shanghai Dongye Technology Co., Ltd., batch number: B20587) (5, 25, 50 mg/kg) [25] and positive drug : silibinin (40 mg/kg) [26] were injected intraperitoneally. The control group and the ethanol liquid diet group were intraperitoneally injected with the same amount of normal saline (0.1 mL/10 g). Four groups of mice were fed alcohol (5 g/kg body weight) by gavage on the last day, and the control group was given isocaloric maltose-dextrin (9 g/kg body weight). Blood and liver samples were collected on day 16 for subsequent experiments.

### 2.5. Cell Culture and Cell Processing

Raw264.7 cells (Shanghai, Chinese Academy of Sciences) were cultured in Dulbecco's modified Eagle's medium (DMEM) medium containing 10% fetal bovine serum in a 37°C, 5% CO_2_ incubator. RAW264.7 cells were then treated with 100 mM ethanol for 12 h, and some cells were treated with ethanol at the same time as AA (40 *μ*M) treatment. Finally, the levels of inflammatory factors and the expressions of intracellular related proteins in the supernatant of RAW264.7 cells were detected.

### 2.6. Detection of ALT, AST, T-CHO, TG, IL-1*β*, IL-6, and TNF-*α*

Mouse serum was extracted by centrifugation at 3000 rpm for 20 min. AST, ALT, TG, and T-CHO kits (Nanjing, China) and ELISA kit (GENEray, Shanghai) were used to detect the corresponding indicators in mouse serum.

### 2.7. Hematoxylin-Eosin (HE) Staining and Oil Red Staining

Livers from C57BL/6J mice were first fixed in 4% paraformaldehyde for 24 h. Sections were then embedded in paraffin and stained with HE and oil red.

### 2.8. Immunohistochemical Staining

Livers from C57BL/6J mice were fixed in 4% paraformaldehyde for 24 h. Paraffin-embedded liver sections (5-*μ*m thick) were deparaffinized and dehydrated, and antigen retrieval was obtained by microwaving in citrate buffer for 15 min. Sections were treated with 0.3% hydrogen peroxide for 15 min, blocked with 2% bovine serum albumin, and then blocked with P-NF-*κ*B-p65 (1 : 200, Bioss, China); incubate overnight with primary antibody. The next day, sections were incubated with the secondary antibody for 60 min at room temperature and stained with 3,3-diaminobenzidine tetrahydrochloride (DAB) for chromogenic detection. Sections were counterstained with hematoxylin solution for 30 s and then dehydrated. Images were finally obtained using a laser scanning confocal microscope (Carl Zeiss, Germany).

### 2.9. Immunofluorescence Staining

RAW264.7 cells were divided into the normal group and model group, first fixed with 4% paraformaldehyde for 30 min and then blocked with 3% BSA for 30 min. The cells in the dish were bound with anti-p-NF-*κ*B-p65 (1 : 200) primary antibody. The samples were incubated with fluorescent secondary antibodies for 1 h in the dark. Nuclei were then stained with DAPI and antifluorescence quencher for 10 min. Images were finally obtained using a laser scanning confocal microscope.

### 2.10. Western Blot Analysis

Proteins were extracted from RAW264.7 cells using radioimmunoprecipitation (RIPA) buffer (Yazyme, Shanghai). Protein concentration was determined by BCA protein detection kit (Biyuntian, Shanghai). SDS-PAGE was used to separate proteins from different samples. After transferring the separated proteins to polyvinylidene fluoride (PVDF) membranes (Bio-Rad, CA, USA), the PVDF membranes were blocked with protein-free fast blocking solution (Yazyme, Shanghai) for 15 min. Blocked PVDF membranes were then incubated in diluted primary antibody for 12 h at 4°C. The dilution ratios of different antibodies (Boaosen, Beijing) were as follows: *β*-actin (1 : 1000), NF-*κ*B-p65 (1 : 1000, Bioss), p-NF-*κ*B-p65 (1 : 1000, Bioss), IL-1*β* (1 : 500, Bioss), IL-6 (1 : 500, Bioss), and TNF-*α* (1 : 500, Bioss). Next, the PVDF membrane and diluted secondary antibody were incubated for 1 h at room temperature. Finally, proteins on PVDF membranes were imaged using enhanced chemiluminescence (ECL) solution (GlpBio, USA), and protein bands were processed using ImageJ to detect protein expression levels.

### 2.11. Real-Time Reverse Transcriptase-Polymerase Chain Reaction (Real-Time RT-PCR) Analysis

Total RNA from RAW264.7 cells and liver tissue were extracted using Trizol LS reagent (Thermo Fisher, Shanghai). After quantification of the extracted RNA, the RNA was reverse transcribed into complementary DNA (cDNA) using the Evo M-MLV Mix Kit (ACCURATE BIOLOGY, China). The mouse gene was amplified by qPCR using the SYBR Green Premix RT-qPCR kit with cDNA as the template. Glyceraldehyde-3-phosphate dehydrogenase (*GAPDH*) was used as an internal reference gene for messenger RNA (mRNA). The primer sequences used in RT-qPCR are shown in Table 1.

### 2.12. Statistical Analysis

All experimental data were analyzed by SPSS software. Statistical significance of pairwise comparisons was analyzed by a *t*-test. Results are expressed as the mean ± standard deviation (*X* ± S.D.), with *P* < 0.05 indicating statistical significance.

## 3. Results

### 3.1. Potential Targets of AA for ASH

**Figure 2:**
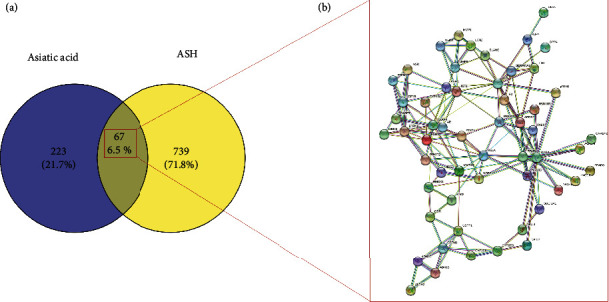
(a) Venn diagram of intersection of asiatic acid and ash targets. (b) Protein interaction network of intersection targets.

We collected the targets of AA using the PharmMapper database and PubMed. After integrating UniProt database entries and eliminating duplicates, 290 targets were obtained. We collected 806 ASH-related targets as disease targets from the GeneCards database. Briefly, 291 active compound targets and 806 disease targets of AA were used to draw a Venn diagram, and 67 overlapping targets were obtained, which were considered the core genes for follow-up research (Figure 2).

### 3.2. Identification and Validation of the Core Targets of AA against ASH

**Figure 3:**
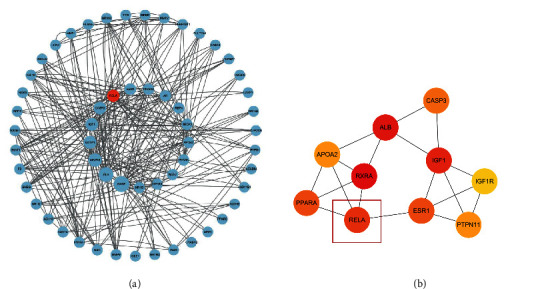
(a) Cytoscape treated protein interaction network. (b) The top ten protein interaction networks obtained by the degree algorithm.

**Figure 4:**
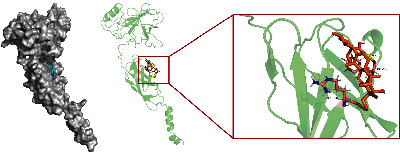
Asiatic acid docking with p65 molecule.

To fully elucidate the potential mechanism of AA in the treatment of ASH, the gene names of AA anti-ASH targets were imported into the String database, and a PPI network was constructed. We plotted the PPI network using the desired interaction score of 0.4 and hid disconnected nodes in the network (Figure 2(b)). To achieve better visualization and identify core targets, we built a network using Cytoscape according to the target degree (Figure 3(a)). Through the degree algorithm, we screened out the top 10 core target-protein interaction networks, and RELA (NF-*κ*B-p65) may be the main target of AA in the treatment of ASH (Figure 3(b)). In addition, we applied molecular docking technology to verify the binding mode of AA and RELA. As shown, AA forms two hydrogen bonds with PHE-239 and ARG-236 in P65 (Figure 4). Binding energies were calculated to assess the degree of complementarity between drug molecules and P65. The free binding energy of AA to P65 is –6.08 kcal/mol. The lower the binding energy, the higher the stability.

### 3.3. Generation of Animal Models and Cell Models [22]

#### 3.3.1. Animal Models

**Figure 5:**
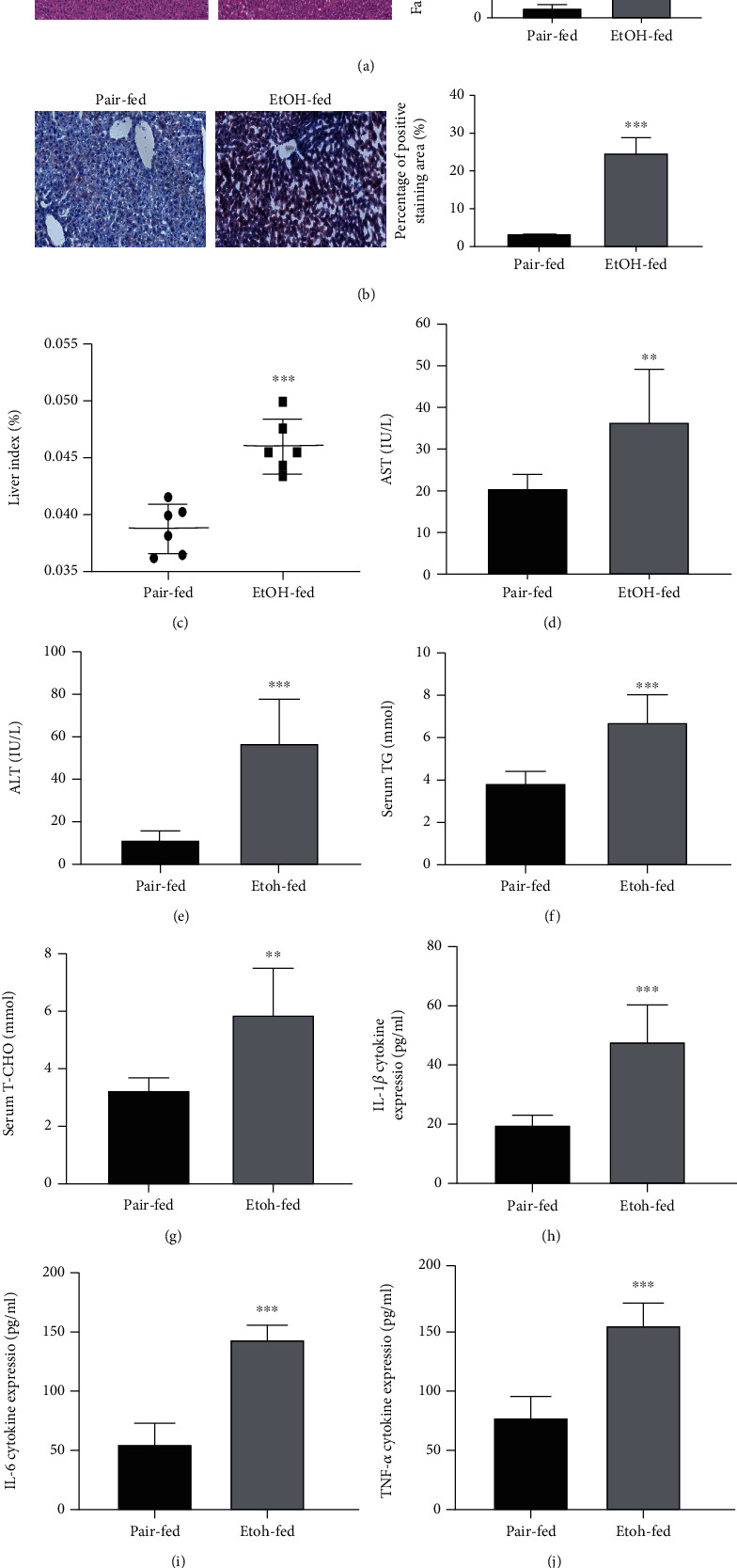
The establishment of animal models. (a) Representative H&E staining of liver sections (original magnification: 200x, scale bar = 50 *μ*m). (b) Representative oil red staining of liver sections (original magnification: 200x, scale bar = 50 *μ*m). (c) Liver index. (d–g) The serum levels of AST, ALT, TG, and T-CHO. (h–j) The serum levels of IL-1*β*, IL-6, and TNF-*α*. ^∗^*P* < 0.05,  ^∗∗^*P* < 0.01, and^∗∗∗^*P* < 0.001 compared with the Pair-fed group. ^#^*P* < 0.05,  ^##^*P* < 0.01, and^###^*P* < 0.001 compared with the EtOH-fed group. The data represent the mean ± S.D. of at least three independent experiments.

By comparing the HE- and oil red-stained pathological sections of the normal group and the model group, we found the ballooning changes of hepatocytes in the tissue slices of the model group, and liver injury and lipid accumulation in the model group were clearly significantly increased compared to the control group (Figures 5(a) and 5(b)). In addition, the liver of the model group was significantly swollen, and the liver weight ratio was higher than that of the normal group (Figure 5(c)). At the same time, the levels of related indices in serum were measured. The levels of AST, ALT, TG, and T-CHO in the serum of the model group were significantly higher than those of the normal group (Figures 5(d)–5(g)), and the cytokine ELISA test results showed that there were more proinflammatory cytokines such as interleukin 1 beta (IL-1*β*), interleukin 6 (IL-6), and tumor necrosis factor alpha (TNF-*α*) in the serum of the model group (Figures 5(h)–5(j)). Based on the above results, we concluded that the mouse model of alcoholic hepatitis was successfully established.

#### 3.3.2. Cell Model

**Figure 6:**
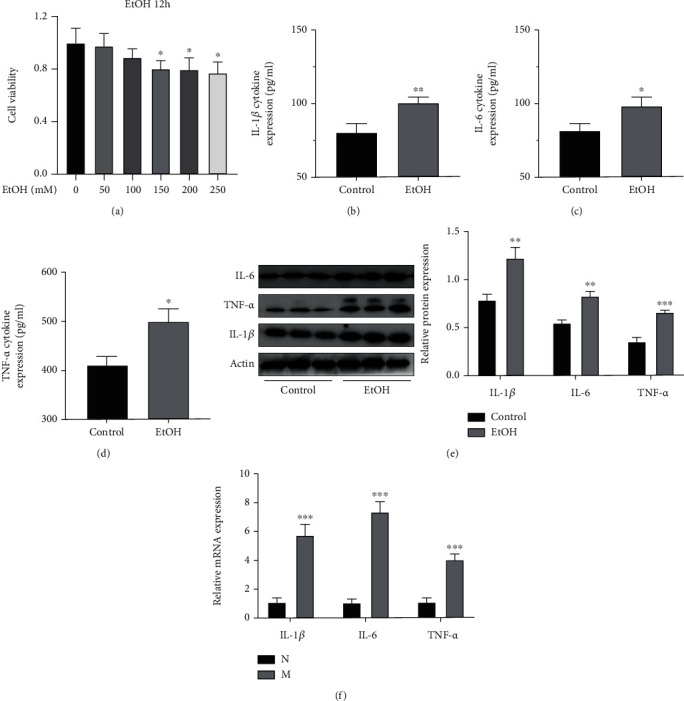
The establishment of cell model. (a) Relative cell viability of RAW264.7 cells. (b–d) Levels of IL-1*β*, IL-6, and TNF-*α* in the supernatant of RAW 264.7 cells. (e) Relative protein expression of IL-1*β*, IL-6, and TNF-*α*. (f) Relative mRNA expression of IL-1*β*, IL-6, and TNF-*α* in the RAW264.7 cells. ^∗^*P* < 0.05,  ^∗∗^*P* < 0.01, and^∗∗∗^*P* < 0.001 compared with the Pair-fed group. ^#^*P* < 0.05,  ^##^*P* < 0.01, and^###^*P* < 0.001 compared with the EtOH-fed group. The data represent the mean ± S.D. of at least three independent experiments.

To further explore the mechanism of action of AA, RAW264.7 cells were stimulated with alcohol to establish an *in vitro* cellular inflammation model. First, the cytotoxicity of alcohol on RAW264.7 cells was detected by Cell Counting Kit-8 (CCK-8), which showed that alcohol concentrations above 100 mM were cytotoxic to RAW264.7 cells and affected cell viability (Figure 6(a)). The microscopy results showed that the shape of RAW264.7 cells changed from circular to pseudo-elongated polygon after being stimulated by 100 mM alcohol. The results of ELISA, RT-qPCR, and Western blot also supported the above results (Figure 6(b)–6(f)). Thus, the RAW264.7 cell inflammation model was successfully established.

### 3.4. AA Alleviates Alcoholic Hepatitis in Mice

**Figure 7:**
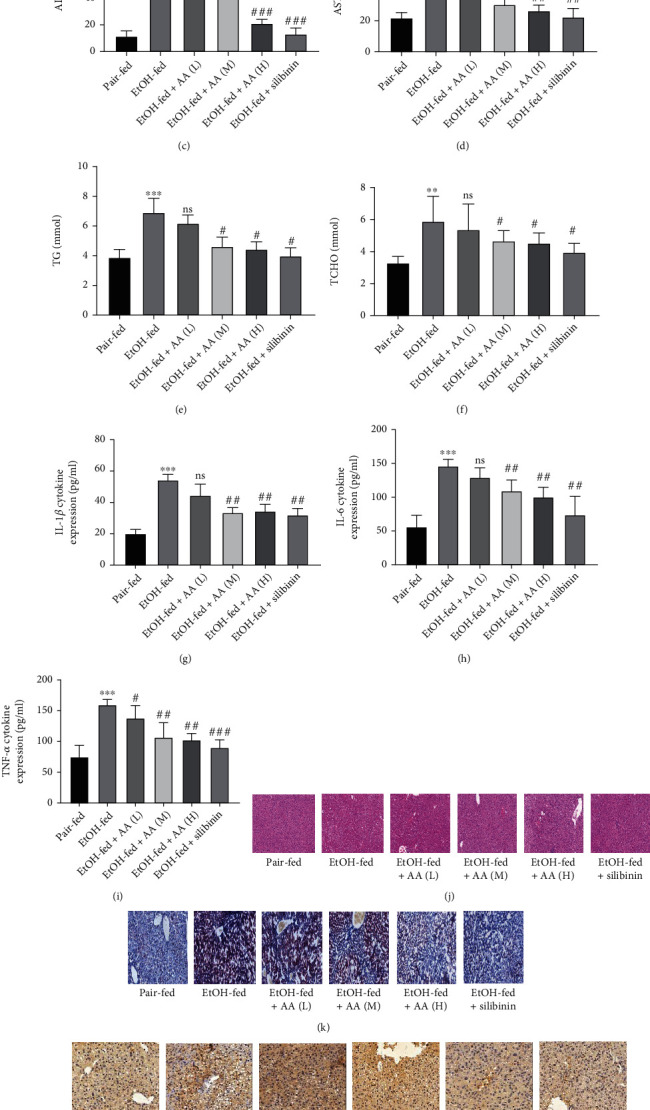
Asiatic acid can relieve alcoholic hepatitis in mice. (a) Body weight and (b) liver index. (c–f) The serum levels of ALT, AST, TG, and T-CHO. (g–i) The serum levels of IL-1*β*, IL-6, and TNF-*α*. (j) Representative H&E staining of liver sections (original magnification: 200x, scale bar = 50 *μ*m). (k) Representative oil red staining of liver sections (original magnification: 200x, scale bar = 50 *μ*m). (l) Representative P-NF-*κ*B-p65 immunohistochemistry staining of liver sections (original magnification: 200x, scale bar = 50 *μ*m). ^∗^*P* < 0.05,  ^∗∗^*P* < 0.01, and^∗∗∗^*P* < 0.001 compared with the control group. ^#^*P* < 0.05,  ^##^*P* < 0.01, and^###^*P* < 0.001 compared with the EtOH group. The data represent the mean ± S.D. of at least three independent experiments.

In terms of body weight, mice in the normal group had fluctuating body weight within the normal range (*x* ± *S*), and the model group and AA group showed a downward trend, but the decrease in the model group was particularly obvious (Figure 7(a)). The liver was obviously inflamed, and the liver weight ratio was significantly higher than that in the normal group (Figure 7(b)). Compared with the model group, the AA group could significantly improve the degree of liver inflammation, and the liver weight ratio was significantly reduced. ALT and AST were the most sensitive indicators of liver parenchymal injury (Figures 7(c) and 7(d)), and TG and T-CHO could effectively reflect the level of lipid accumulation (Figures 7(e) and 7(f)). The examination results showed that AST, ALT, TG, and T-CHO in the model group were significantly higher than those in the normal group, while those in the AA and silibinin-administration groups had decreased. In addition, serum IL-1*β*, IL-IL-1*β*, IL-6, and TNF-*α* levels were measured with ELISA kits. The results showed that the levels of IL-1*β*, IL-6, and TNF-*α* were increased after AA and silybinin treatment. The level was significantly lower than that of the model group (Figures 7(g)–7(i)). The findings showed that AA attenuated liver damage, lipid accumulation, and inflammatory responses in mice. The results of immunohistochemical staining of mouse liver for HE, oil red, and NF-*κ*B-p65 are shown (Figures 7(j)–7(l)). The model group showed typical characteristics of alcoholic hepatitis, including swelling of hepatocytes, loose cytoplasm, clusters of microfilaments, typical balloon degeneration of hepatocytes, large vesicular steatosis, inflammatory cell infiltration, and cell necrosis, but no abnormality was found in normal mice. The above-mentioned liver injury, inflammatory cell infiltration, and lipid accumulation were significantly reduced in the AA group and the silibinin-treated group, but mild liver injury and vesicular steatosis were still seen compared to the normal group. The results of immunohistochemistry showed that the expression of NF-*κ*B-p65 protein in the liver of the model group was significantly higher than that of the normal group, but was significantly inhibited in the AA group. The comparison of the three dosing groups showed that AA may alleviate alcoholic hepatitis by inhibiting the expression of NF-*κ*B.

### 3.5. Asiatic Acid in RAW264.7 Cells May Inhibit Alcohol-Induced Inflammatory Response by Regulating NF-*κ*B

**Figure 8:**
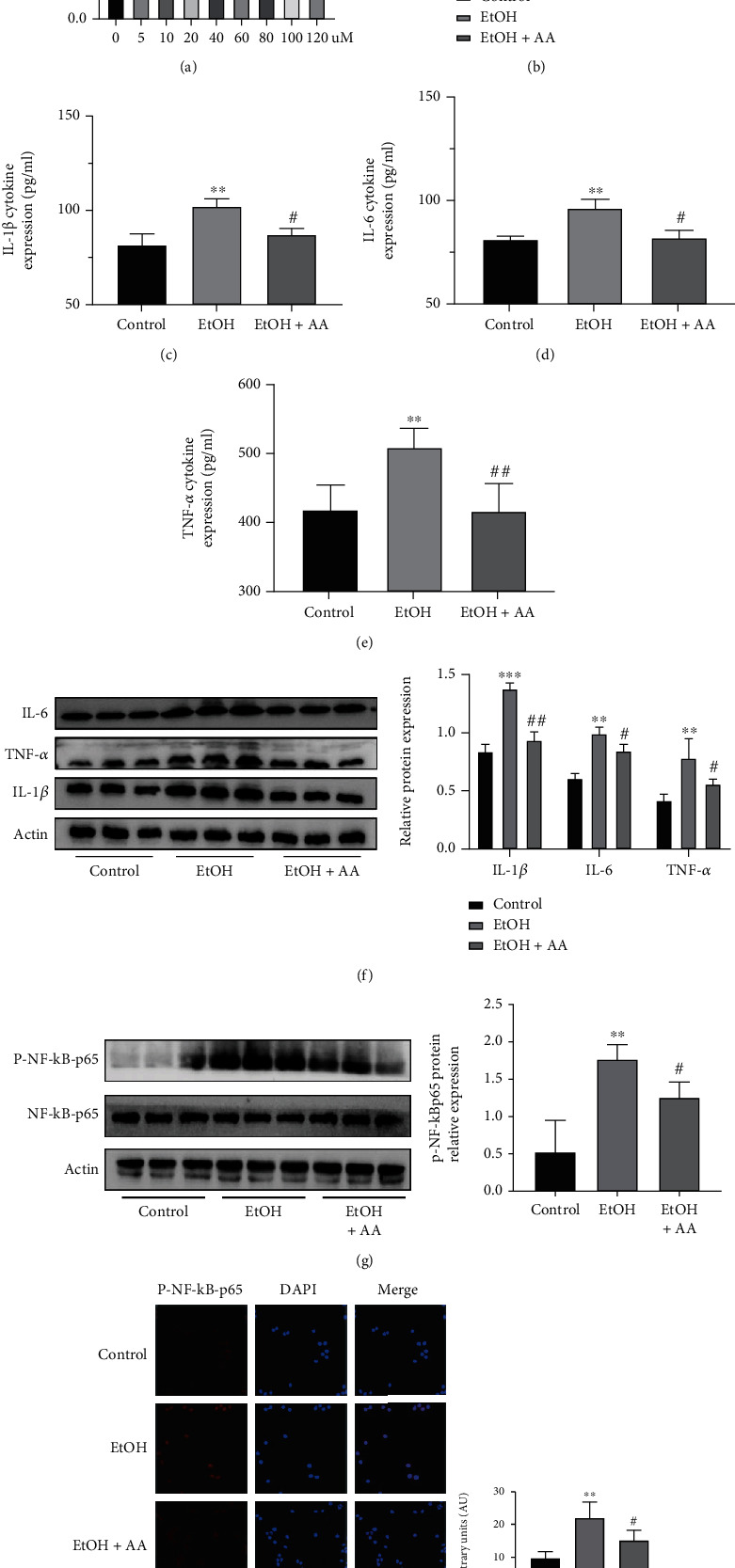
Asiatic acid may inhibit alcohol-induced inflammation by regulating NF-*κ*B pathway in the RAW264.7 cells. (a) Relative cell viability of RAW264.7 cells. (b) Relative mRNA expression of IL-1*β*, IL-6, and TNF-*α* in the RAW264.7 cells. (c–e) Levels of f IL-1*β*, IL-6, and TNF-*α* in the supernatant of RAW 264.7 cells. (f) Relative protein expression of IL-1*β*, IL-6, and TNF-*α* in the RAW264.7 cells. (g) Relative protein expression of P-NF-*κ*B-p65 in the RAW264.7 cells. (h) Representative immunofluorescence images of P-NF-*κ*B-p65 (red) (×200) in the control and EtOH groups are presented (scale bar = 20 *μ*m). ^∗^*P* < 0.05,  ^∗∗^*P* < 0.01, and^∗∗∗^*P* < 0.001 compared with the Control group. ^#^*P* < 0.05,  ^##^*P* < 0.01, and^###^*P* < 0.001 compared with the EtOH group. The data represent the mean ± S.D. of at least three independent experiments.

To further explore the mechanism of AA regulating inflammation, we established an *in vitro* inflammation model of RAW264.7 cells using alcohol stimulation (100 mM). Then, we used the CCK8 assay to detect the cytotoxicity of AA in RAW264.7 cells and determined the cellular administration concentration of AA; to further explore the mechanism of AA regulating inflammation, we established an *in vitro* inflammation model of RAW264.7 cells using alcohol stimulation (100 mM). Then, we used the CCK8 assay to detect the cytotoxicity of AA in RAW264.7 cells and determined the cellular administration concentration of AA (40 *μ*M) (Figure 8(a)). After alcohol-stimulated RAW264.7 cells were simultaneously treated with AA, analysis by RT-qPCR, ELISA, and Western blot showed that the levels of IL-1*β*, IL-6, and TNF in the AA group were significantly higher than in the alcohol-stimulated group (Figures 8(b)–8(f)). Western blot and P-NF-*κ*B-p65 cell immunofluorescence results showed that the phosphorylation level of the highly expressed NF-*κ*B-Pp65 in the alcohol-stimulated group was significantly decreased after administration of AA (Figures 8(g) and 8(h)). These results suggest that the administration of AA to alcohol-stimulated RAW264.7 cells may inhibit alcohol-induced cellular inflammatory response by inhibiting the phosphorylation of NF-*κ*B-p65.

## 4. Discussion

Alcoholic hepatitis is an acute inflammatory liver disease with high short- and long-term morbidity and mortality. Due to insufficient effective drug treatments for ASH and alcohol dependence, the development of new effective treatments is imperative. In a recent literature, natural plant compounds have been frequently reported for the treatment and inhibition of alcoholic hepatitis. AA extracted from the plant *Centella asiatica* has been previously noted for its anti-inflammatory, wound healing, antidiabetic, antioxidant, and hepatoprotective properties [9, 10, 27]. There have been many literature reports on the anti-inflammatory properties of AA. For example, studies have found that AA has a positive inhibitory effect on lipopolysaccharide-induced lung inflammation and lipopolysaccharide-induced endometrial epithelial cell inflammation [25, 28]. AA was found to reduce LPS-induced inducible nitric oxide synthase (iNOS), cyclooxygenase 2 (COX-2), IL-6, and IL-1*β* in naive 264.7 cells [12]. In addition, AA was found to improve metabolic and blood metabolic abnormalities in rats caused by metabolic syndrome (MS) by reducing oxidative stress and inflammation [29]. In addition, many studies have shown that AA can protect the liver from damage through mechanisms against mitochondrial stress and cellular antioxidant systems. AA can inhibit liver fibrosis by blocking the TGF-*β*/Smad pathway [30]. AA attenuates ethanol-induced liver injury by inhibiting oxidative stress and Kupffer cell activation [31]. As AA has marked anti-inflammatory and hepatoprotective properties, the question remains whether it also has good performance on ASH.

In the early stage of this study, we found that AA could inhibit the occurrence and progression of alcoholic hepatitis in mice through liver section staining and mouse liver serum biochemistry. As previously described, AA inhibited the development of alcoholic hepatitis in mice and reduced lipid droplet aggregation, inflammatory cell infiltration, and cell necrosis. Previous studies have shown that inflammation is key to the development of alcoholic liver disease, and anti-inflammatory treatment can effectively block the development of alcoholic liver disease [2, 6, 32]. NF-*κ*B includes a family of nuclear transcription factors and is a major inflammatory pathway that is activated during the development of liver injury [14, 33]. Further mechanistic studies revealed that AA may ameliorate the symptoms of alcoholic hepatitis in mice by inhibiting the phosphorylation of NF-*κ*B-p65 and reducing its expression. In addition, experimental studies suggest that AA can effectively inhibit DNA binding activity and protein expression of NF-*κ*B-Pp65, reduce the level of pro-inflammatory cytokines, and inhibit the inflammatory response in animal and cell experiments.

**Figure 9:**
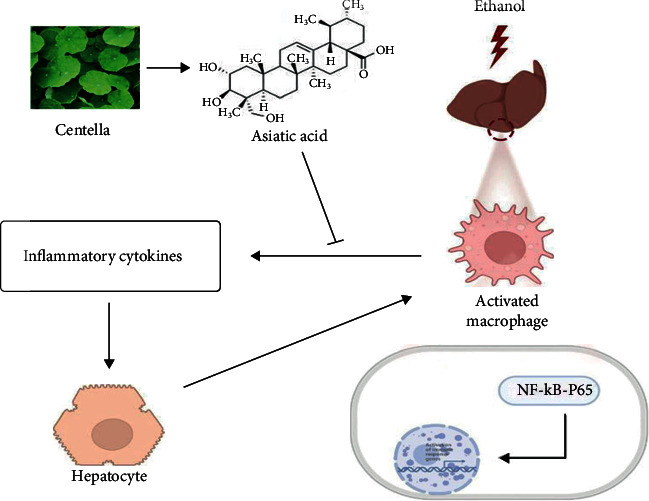
Mechanism of asiatic acid alleviating alcoholic steatohepatitis through the NF-*κ*B signaling pathway.

In summary, this study combined network pharmacology with *in vitro* and *in vivo* experiments to verify the effect of AA on ASH. The mechanism diagram of this study is shown in (Figure 9).

The present findings provide evidence that AA plays a key role in the protection of alcoholic hepatitis and effective mitigation of liver injury and inflammatory responses during the development of alcoholic hepatitis through the NF-*κ*B pathway. Therefore, AA is promising in the treatment of alcoholic hepatitis and merits further study.

## Figures and Tables

**Table 1:** RT-qPCR primer sequence.

Gene	Forward	Reverse
GAPDH	AGGTCGGTGTGAACGGATTTG	TGTAGACCATGTAGTTGAGGTCA
IL-1*β*	GCAACTGTTCCTGAACTCAACT	ATCTTTTGGGGTCCGTCAACT
IL-6	TAGTCCTTCCTACCCCAATTTCC	TTGGTCCTTAGCCACTCCTTC
TNF-*α*	GACGTGGAACTGGCAGAAGAG	TTGGTGGTTTGTGAGTGTGAG

## Data Availability

The data used to support the findings of this study are available from the corresponding author upon request.
